# Prof. John Hugh McQuillen

**Published:** 1879-04

**Authors:** 


### OBITUARY.

[*Advance Sheets kindly furnished by Dr. J. W. White, Editor of the* DENTAL COSMOS.]

*Prof. John Hugh McQuillen, M. D., D. D. S.*—Suddenly, on March 3, 1879, Dr. John H. McQuillen, in the fifty-fourth year of his age.

Dr. McQuillen was born in Philadelphia, February 12, 1826. He was the son of Captain Hugh McQuillen, who served under Decatur during the war of 1812. The ancestors of his mother, Martha Scattergood, came to America with William Penn. He received his early education in the Friends' schools in Philadelphia, and at the age of sixteen entered as clerk in an importing house with the purpose of devoting himself to commercial pursuits. His tastes, however, inclined him to medicine, and after attaining his majority he commenced its study in the Jefferson Medical College, from which he graduated in 1852. Meantime he was also studying dentistry, and in 1853 he received the honorary degree of D. D. S. at the first commencement of the Philadelphia College of Dental Surgery. In 1857 he was elected to the chair of operative dentistry and dental physiology in the Pennsylvania College of Dental Surgery, holding that position for one year, and relinquishing it to take the chair of general anatomy and physiology, which he resigned in 1862. In 1863, principally through the efforts of Dr. McQuillen, a charter was obtained for the Philadelphia Dental College, of which institution he was made dean and professor of physiology, retaining both positions until his death. In 1859 the publication of the *Dental Cosmos* was commenced, Dr. McQuillen becoming one of its editors, which relation was continued until his retirement in 1871.

Dr. McQuillen has been a frequent contributor to the literature of the profession from his first connection with it, and has held important positions in various dental societies, including the presidency of the American Dental Association, of the Pennsylvania State Dental Society, and of the Odontographic Society of Pennsylvania.

As an operator Dr. McQuillen was recognized as possessing more than average skill, supplemented by an earnestness and conscientiousness which were a guarantee of faithful service.

As a teacher he was enthusiastic and ambitious, laboring to the best of his ability to prepare those whom he instructed for the intelligent and successful practice of their vocation. Hundreds of the alumni of the institutions in which he taught will hear of his decease with sincere sorrow.

Dr. Mc.Quillen was by nature an organizer. To his organizing faculty, as well as to his zeal, energy, and tact, the success of the Philadelphia Dental College is largely due, and it was these qualities which caused him to be recognized as a power in the profession at large. If ever any man was consecrated to the chosen work of his life, John H. McQuillen was. He devoted to the original organization of the American Dental Association, to its development and to the general educational interests of the dental profession, the best efforts of his life. Other men may be found of greater natural gifts, of larger attainments, and of more brilliancy, but none who have contributed more freely, more constantly, more unselfishly to the general good. And though he possessed personal gifts and graces which won the respect and esteem of a large circle of friends, professional and otherwise, who will hold his memory in affectionate remembrance; though he was courteous, genial, and kindly in spirit and manner; though he was hospitable to a fault, it was not to these qualities chiefly that he owed his position and his usefulness in the profession, but to a life devoted to its improvement and elevation. Considering his energy, his industry, his unselfishness, the worthiness of his aims, the work he has done, the influence of his example as an advocate of educational progress and reform in his profession,—an influence which will be felt for good through many years to come,—the sudden termination of his earthly career is a calamity, not alone to his family and personal friends, not alone to the institution of which he was the recognized head, but to the practitioners of dentistry wherever located, and especially to American dentists. His place will be difficult to fill; perhaps not in this generation will it be in all respects completely filled.

The sad side of the history is that he allowed his interest in the school with which he was associated, and the general interests of the profession as an organized body, to make exhausting and damaging drafts upon him, involving the sacrifice of time, money, practice, ease, and pleasure. Had he brought the same ability, the same industry, the same energy, the same concentration, the same persistence to the accumulation of means by the practice of his profession, he might have left his family handsomely provided for. That he did not do so not only constitutes a cause of regret for their sakes, but calls for a substantial recognition of his valuable labors and services in the advancement of the dental profession in usefulness, self-respect, and public regard, and in strengthening fraternal courtesy and co-operation among its members.

Dr. McQuillen leaves a widow and four children, one of whom, Dr. Daniel Neall McQuillen, a graduate of the Philadelphia Dental College, has but recently engaged in practice. Mrs. McQuillen will receive the hearty sympathy of hundreds in the profession who have shared the hospitality of her home, and who will remember with what interest and kindness she received and ministered to her husband's friends.

The resolutions appended show the estimate in which Dr. McQuillen was held in his own city, and by his colleagues and pupils.

At a meeting of the dental profession, held at the Philadelphia Dental College, March 5, called with reference to the decease of Dr. John H. McQuillen, the following preamble and resolutions were adopted by a rising vote:

*Whereas*, Dr. J. H. McQuillen has been suddenly removed from among us by death; it becomes our privilege as well as our sad duty to make record of the event, and to express our estimate of his worth and our sense of the loss thus sustained; therefore:

*Resolved*, That Dr. McQuillen has been for many years so identified with the interests of the dental profession, and so earnest in their advancement, so indefatigable in his efforts in behalf of the elevation of the standard of education, of graduation, and of practice, that his death leaves no one man who in all respects fills the place thus made vacant.

That while as an operator he was gifted with more than ordinary ability; while as a teacher he was conscientious and earnest, his labors in the organization of the profession and in promoting its educational interests gave him marked preeminence.

That, more ready to serve than to be served, more solicitous for the advancement of the profession with which he was identified than for personal advantage, he, indeed too often, ignored the latter in the effort to promote the former.

That a life devoted to the self-sacrificing service of his profession, and to the promotion of all plans having for their object its improvement, made him an example of concentration, of industry, and of persistent effort worthy of imitation.

That, while thus recording our appreciation of his professional attainments and labors, we desire also to express our affectionate remembrance of him as a genial, generous, sympathetic gentleman, and to tender to his bereaved family the assurance of our profound sympathy.

---

At a special meeting of the Faculty of the Philadelphia Dental College, held on Wednesday, March 5, 1879, the following preamble and resolutions were offered, and, after appropriate remarks by members of the Faculty, were adopted:

*Whereas*, The sad intelligence has reached us of the sudden death of Dr. John H. McQuillen, practically the founder of this institution, and its Dean since its establishment, sixteen years ago; and,

*Whereas*, It is fitting that we should place on record a testimonial of our high appreciation of his ability, industry, untiring zeal, and steadfast consecration to the interests ofthis school; therefore,

*Resolved*, That to his organizing faculty, his earnest efforts, and to his unselfish devotion to its interests, the Philadelphia Dental College owes to an extent not to be estimated, its successful career as an educational institution, and its high reputation both at home and abroad: trials, impediments, opposition—circumstances which operated as discouragements to others—only serving to stimulate him to fresh endeavor.

That in his death the dental profession has lost one who has done as much perhaps as any one man to elevate the standard of dental education and practice in this city, in this country, and to no little extent throughout the world; his consecration to the furtherance of all efforts which in his judgment would advance the interests of the dental profession having so won the confidence of his brethren as to command their recognition of him as an able and trustworthy leader.

That, appreciating his talents, his attainments, his labors, his earnestness and his self-sacrificing spirit in all that concerned the interests of this school, we especially cherish the memory of his numerous manifestations of personal interest and kindly helpfulness.

That, with a copy of this expression of our appreciation, esteem, and affection, we tender to his widow and children our sincere sympathy.

---

At a meeting of the Alumni Association of the Philadelphia Dental College, held March 5th, 1879, the following preamble and resolutions were adopted:

*Whereas*, We have received the painful intelligence of the decease of our beloved and honored professor, Dr. J. H. McQuillen; therefore,

*Resolved*, That in his death the Philadelphia Dental College has lost one who was ever ready to devote his time, talents, and best efforts to the promotion of its highest interests.

That the profession has lost an earnest worker in the cause of dental education—one to whose indomitable energy and perseverance the college was indebted for its success, in which every graduate and student feels a legitimate pride.

That a copy of these resolutions be forwarded to his bereaved family, with the expression of our sincere sorrow and sympathy.

---

At a meeting of the students of the Philadelphia Dental College Wednesday morning, March 5, 1879, the following resolutions were adopted:

*Whereas*, Our friend and beloved teacher, Dr. J. H. McQuillen, has been removed by death ; it is hereby

*Resolved*, That we receive the intelligence of his sudden decease with deep regret and heartfelt sorrow.

That in his death the Philadelphia Dental College loses a most able instructor, the dental profession an arduous and conscientious worker, and society a useful and honored member.

That a copy of these resolutions be sent to the family of the deceased with the assurance of our deep sympathy in their bereavement, also that a copy be furnished the *Dental Cosmos* and the city papers.

